# Dietary management of gestational diabetes: A review

**DOI:** 10.1097/MD.0000000000038715

**Published:** 2024-07-12

**Authors:** Bin-Yang Long, Xin Liang

**Affiliations:** a School of Medicine and Life Sciences, Chengdu University of Traditional Chinese Medicine, Chengdu, China; b Reproductive Maternity and Childhood Hospital Affiliated to Chengdu University of Traditional Chinese Medicine, Chengdu, China.

**Keywords:** diet, GDM, gestational diabetes, pregnant women

## Abstract

Gestational diabetes mellitus (GDM) is a common condition in pregnant women that can affect the health of both the mother and the fetus. A healthy diet reduces the risk of GDM, while on the contrary, an unhealthy diet can increase the risk of developing GDM. Dietary interventions remain an important way to control GDM at this time. However, real-life diets are complex and varied, and the effect of these diets on gestational diabetes is unknown. This article summarizes research related to dietary control of GDM. Hopefully, this will help with dietary interventions for people with GDM.

## 1. Introduction

Gestational diabetes is diabetes first diagnosed in the second or third trimester of pregnancy.^[1]^ Gestational diabetes increases the risk of long-term complications for both mother and fetus, including obesity, impaired glucose metabolism and cardiovascular disease.^[2,3]^ Dietary modifications and increased physical activity are the mainstay of treatment for gestational diabetes mellitus (GDM).^[2,4]^ A healthy diet reduces the risk of GDM, and conversely, an unhealthy diet can increase the risk and harm of developing GDM. Deficiencies of certain micronutrients are also strongly associated with the development of diseases during pregnancy, iodine^[5,6]^ and iron^[7]^ deficiencies are common in pregnant women during pregnancy. Lodine deficiency can lead to poorer than normal fetal brain development,^[8]^and iron deficiency can lead to maternal and fetal anemia and intrauterine hypoxia.^[7]^This shows that dietary management during pregnancy is very important, and a proper diet can reduce the risk of illness for both the pregnant woman and the fetus. Dietary interventions remain an important way to control GDM at this time. However, real-life diets are complex and varied, and the effect of these diets on gestational diabetes is unknown. This article summarizes research related to dietary control of GDM. We hope to find dietary combinations that are suitable for people with GDM and people at high risk of GDM to treat and prevent GDM.

## 2. Polyunsaturated fatty acid

Docosahexaenoic acid (DHA) comes mainly from food. DHA is widely believed to affect the neurodevelopment of fetuses and babies, and is often taken by pregnant women to promote the brain development of the babies they give birth to.^[9]^ Some studies have shown that DHA does not prevent GDM.^[10–15]^ Some studies have shown that DHA can prevent GDM.^[16–18]^ Meanwhile, prospective clinical studies have shown that DHA increases the risk of GDM.^[19,20]^ Then, pregnant women with established GDM can reduce blood glucose, lipids, high-sensitivity C-reactive protein and insulin resistance levels by consuming DHA.^[21,22]^

Eicosapentaenoic acid (EPA) comes mainly from food. EPA is a type of polyunsaturated fatty acid, EPA helps reduce cardiovascular disease.^[23,24]^ Some studies suggest EPA is ineffective in preventing GDM.^[11,14,15]^ Some studies have shown that EPA can prevent GDM.^[17,18]^Pregnant women with established GDM can reduce blood glucose, lipids, high-sensitivity C-reactive protein and insulin resistance levels by consuming EPA.^[21,22,25]^

## 3. Total fat and saturated fatty acids

Elevated serum palmitic acid levels are strongly associated with GDM. The proportion of plasma palmitic acid has been found to be positively correlated with GDM, whereas plasma stearic acid, arachidonic acid, behenic acid, and lignoceric acid have been found to be inversely correlated with GDM.^[26]^ Similarly, other studies have shown that elevated serum palmitic acid levels are strongly associated with GDM.^[27–29]^ In addition, not only palmitic acid, but the total percentage of serum saturated fatty acids increased and the total percentage of unsaturated fatty acids decreased in patients with GDM.^[30]^

Clinical studies and animal experiments have shown that, total fat, including triglycerides and cholesterol, is an independent risk factor for adverse pregnancy outcomes in patients with GDM.^[29,31–34]^ Some studies suggest that a diet high in total fat and saturated fatty acids may increase the risk of developing GDM.^[35–39]^ Polyunsaturated fatty acids and monounsaturated fatty acids in place of saturated fatty acids are associated with a reduced risk of developing GDM.^[40]^ High-cholesterol dietary patterns are rich in meat and eggs from livestock and poultry, but low in grains.^[38,39]^

## 4. Dietary fibers

Dietary fiber could reduce GDM development through gut flora-short-chain fatty acid-placental inflammation axis in GDM mouse model.^[41]^ Clinical studies have also shown that a high dietary fiber diet (15–30 g/d) is an important tool for improving all types of diabetes, including GDM.^[42]^ Dietary supplementation with blueberries and soluble fiber (280 g of whole blueberries and soluble fiber 12 g/d) reduced the risk of GDM in obese women in a randomized controlled trial.^[43]^ Other studies have also shown that a diet high in dietary fiber reduces the risk of developing GDM.^[44–46]^ It has also been shown that dietary fiber supplementation during pregnancy in Chinese women of advanced maternal age does not reduce the incidence of GDM, but improves glucose metabolism, gestational weight gain, and preterm delivery.^[47]^ Although a diet high in dietary fiber can help prevent and treat GDM, it is inconsistent in its requirements, including 9.5 g dietary fiber/d,^[48]^ Consume one cup of whole berries and one cup of leafy greens per day,^[49]^ 24 g dietary fiber/d.^[50]^ Beans, fruits, whole grains and vegetables rich in dietary fiber may reduce the risk of GDM,^[51–53]^ whereas excess consumption of fruits, potatoes and fruit juices is associated with an increased risk of GDM.^[54]^

## 5. Carbohydrates

It has been suggested that although a high-quality carbohydrate diet is not very useful in the treatment of GDM, it can reduce the amount of insulin used.^[55,56]^ It has also been suggested that a high-quality carbohydrate diet significantly reduces 2-hour postprandial glucose in GDM patients, but has no significant effect on fasting glucose and insulin requirements.^[57]^ Other studies have shown that a high-quality carbohydrate diet reduces the chances of gestational weight gain and preterm labor in women at high risk of GDM, but does not prevent GDM in these women.^[58]^

## 6. Vitamin

Vitamin D reduce insulin resistance, obesity, macrovascular microvascular damage in type 2 diabetes, insulin autoimmunity, GDM, and maintain oxidative-peroxidative balance.^[59]^ Vitamin D deficiency not only puts pregnant women at increased risk for pre-eclampsia, gestational diabetes, and preterm labor, but also puts infants at increased risk for low birth weight, decreased bone mass, bronchitis, asthma, type 1 diabetes, multiple sclerosis, and autism.^[60–62]^ Meta-analyses have also shown that vitamin D deficiency is associated with GDM.^[63–68]^ Analysis of clinical data also suggests that vitamin D insufficiency in early pregnancy is significantly associated with an increased risk of GDM.^[69]^ A clinical randomized controlled trial showed that supplementation with both vitamin D and inositol significantly reduced the risk of GDM.^[70]^ However, some studies suggest that vitamin D deficiency in early pregnancy does not lead to GDM.^[71–74]^ A randomized controlled trial noted that vitamin D supplementation during pregnancy in vitamin D-deficient patients did not prevent the development of PIH and GDM.^[75]^ Vitamin D deficiency not associated with GDM in women undergoing assisted reproductive technology.^[76]^

A Chinese prospective cohort noted that high levels of folic acid and vitamin B12 supplementation in early pregnancy are associated with an increased risk of GDM.^[77]^ Another systematic review and meta-analysis in China indicated that there was no association between maternal vitamin B12 concentrations and GDM in the first trimester, but low vitamin B12 concentrations in the second or third trimester were associated with an increased risk of GDM.^[78]^ A prospective study showed that serum vitamin B12 levels were not associated with GDM but were inversely related to fasting glucose concentrations.^[79]^ Retrospective Cohort Studies Suggest Serum Folate Excess or Vitamin B12 Deficiency May Lead to Increased Risk of GDM.^[80,81]^

Low vitamin B6 can also cause GDM.^[82]^ Animal studies have shown that vitamin B6-deficient chow feeding results in hyperglycemia and glucose intolerance in C57BL/6J pregnant mice, but not in DBA/2J mice.^[83,84]^

A retrospective cohort study in China suggests that low vitamin E levels in early pregnancy and decreased vitamin E levels during pregnancy are associated with an increased risk of GDM.^[85]^ Another systematic evaluation and meta-analysis also showed that vitamin E levels were significantly lower in pregnant women with GDM than in regular pregnant women, but there was no difference in vitamin E levels between overweight pregnant women with GDM and regular pregnant women.^[86]^ Clinical randomized controlled trial demonstrates that zinc and vitamin E supplementation in GDM patients significantly improves insulin metabolism, lipid distribution, and gene expression levels of ppa-PAP and LDLS.^[87–89]^ Results from animal studies also support that vitamin E supplementation may be beneficial in GDM.^[90]^ However, other clinical studies have concluded that high vitamin E levels lead to GDM.^[91,92]^

The results of prospective studies suggest that increased intake of vitamin A from animal foods is associated with a reduced risk of GDM and that there is no association between plant vitamin A intake and GDM.^[93]^ Other prospective studies have come to the opposite conclusion, suggesting that high vitamin A levels lead to GDM.^[94]^

## 7. Trace element

A study suggests that micronutrient supplementation during pregnancy is beneficial for patients with GDM.^[95,96]^ Meta-analyses have shown that high hemoglobin or ferritin increases the risk of GDM.^[97,98]^ Some prospective studies showed that long-term iron supplementation during pregnancy increased the risk of GDM.^[99–105]^ Reduced plasma magnesium due to high-dose iron supplementation may contribute to the development of GDM.^[106]^

Low serum selenium levels may contribute to the development of GDM, and selenium supplementation may reduce the risk of GDM.^[107–112]^

Meta-analyses suggest that magnesium deficiency contributes to GDM.^[113–115]^ Prospective studies have also shown that magnesium deficiency contributes to the development of GDM.^[106]^ However, other studies have shown that magnesium is not associated with the development of GDM.^[116,117]^

## 8. Others

Higher intake of high-sugar beverages before pregnancy is an independent risk factor for GDM.^[118]^

Caffeine intake in the first trimester of pregnancy was not associated with the risk of GDM, and heavy cola consumption led to GDM.^[119]^

There is no evidence to support an association between maternal smoking during pregnancy and the risk of GDM.^[120]^

Higher consumption of fast food (burgers, sausages and pizza) before pregnancy is an independent risk factor for GDM.^[121]^

Women who reported very fast eating speed, compared with those reporting slow eating speed, were associated with an increased incidence of GDM, which may be largely mediated by increased body fat.^[122]^

New cravings in the first trimester of pregnancy were associated with dietary intake. Craving salty foods may predict reduced risk of developing GDM, whereas craving sweet food does not appear to alter one’s risk.^[123]^

## 9. Conclusions

For polyunsaturated fatty acids, the role of DHA in GDM is controversial, but EPA is favorable and harmless for GDM. Serum palmitic acid levels are positively associated with the risk of GDM, and an increase in the proportion of triglycerides, cholesterol and saturated fatty acids in the diet increases the risk of GDM. The results of almost all studies show that dietary fiber prevents GDM. high-quality carbohydrates have little effect on GDM, but appear to reduce insulin use and improve complications in patients with GDM. Vitamin D and vitamin B6 supplementation appear to treat and prevent GDM, vitamin E supplementation is beneficial for patients with GDM but excessively high levels of vitamin E may contribute to GDM, and the role of vitamin A, folic acid, and vitamin B12 in GDM is controversial. Reduced iron intake, selenium and magnesium supplementation reduce the risk of GDM. High-sugar beverages, colas, fast food, rapid eating and craving for sweets may increase the risk of GDM, while caffeine and smoking do not seem to be associated with GDM.

## Author contributions

**Data curation:** Binyang Long.

**Funding acquisition:** Xin Liang.

**Investigation:** Binyang Long.

**Methodology:** Binyang Long.

**Software:** Binyang Long.

**Supervision:** Xin Liang.

**Writing – original draft:** Binyang Long.

**Writing – review & editing:** Binyang Long.
